# Room Temperature Electrodeposition of Ready-to-Use TiO_x_ for Uniform p-n Heterojunction Over Nanoarchitecture

**DOI:** 10.3389/fchem.2022.832342

**Published:** 2022-02-22

**Authors:** Yufeng Cao, Huajian Qiao, Yalong Zou, Na An, Yang Zhou, Deyu Liu, Yongbo Kuang

**Affiliations:** ^1^ Ningbo Institute of Materials Technology and Engineering, Chinese Academy of Sciences, Ningbo, China; ^2^ Center of Materials Science and Optoelectronics Engineering, University of Chinese Academy of Sciences, Beijing, China

**Keywords:** titanium oxide, electrochemical deposition, heterogeneous nanostructure, cuprous oxide photocathode, p-n junction

## Abstract

The photocathodes are essential in photoelectrochemical systems for harvesting solar energy as green fuels. However, the light-absorbing p-type semiconductor in them usually suffers from carrier recombination issues. An effective strategy to address it is fabricating the p-n heterojunction to create an interfacial electric field. However, plenty of deposition process of the n-type layer for this purpose requires either sophisticated instruments or subsequent treatments, which may damage the vulnerable p-type structure. Herein, we report a mild approach for a ready-to-use n-type layer with full functionality. Structural analyses proved the successful coating of a uniform titania layer (up to 40 nm) over Cu_2_O without damaging its structure. Owing to the high Ti^3+^ content, the layer possesses excellent charge transport ability and requires no additional annealing. The heterojunction effectively facilitates the carrier separation and positively shifts the photocurrent onset potential for 0.2 V. The Mott–Schottky plot and the impedance study reveal an enhanced carrier collection with reduced charge transfer resistances. Such a nano-heterojunction can be further loaded with the hydrogen evolution catalyst, which almost doubles the photocurrent with an extended lifetime than that of the pristine Cu_2_O nanoarray. This approach puts forward a potentially scalable and efficient choice for fabricating photoelectrochemical devices.

## Introduction

Oxide photocathodes have attracted great attention for the photoelectrocatalytic production of hydrogen fuels by solar water splitting (Li et al., 2020). Particularly, Cu_2_O has been demonstrated as one of the most promising choices among many light-absorbing materials. The intrinsic Cu vacancies as shallow acceptor levels lead to decent p-type characteristics (Olsen et al., 1982). However, challenges are limiting the performance of photocathodes. Specifically, many photoelectrodes suffer from surface redox instability and self-reduction by the interfacial accumulation of photoelectrons (Tilley et al., 2014). More importantly, the unsatisfactory charge separation and internal charge transport strongly degrade the practical efficiency from the theoretical expectation (Wick and Tilley, 2015; Luo et al., 2016). The short diffusion length of charge carriers in Cu_2_O restricted the thorough extraction of photoelectrons (Musselman et al., 2010). It has been demonstrated that various eye-of-sight deposition techniques can be used for the Cu_2_O structure with minor curvatures to address these challenges (Paracchino et al., 2012; Minguez-Bacho et al., 2015), such as the thermal evaporation or e-beam deposition, providing a straightforward and effective solution to load diverse functional layers (Dubale et al., 2015; Han et al., 2015). The multilayered integration of desired components greatly enhances both the efficiency and stability of Cu_2_O photocathodes (Zhang et al., 2013; João et al., 2016). For instance, using the rationally designed Ga_2_O_3_/TiO_2_/RuO_2_ overlayer, the photocathode exhibited an unprecedented stable high internal quantum efficiency over 120 h (Pan et al., 2018).

On the other hand, in recent years, the morphology control of the p-type light-absorbing layer has been recognized as another answer to effectively balance the surface area and charge transport pathways (Chen et al., 2012; Concina et al., 2017). The state-of-the-art efficiency has been achieved by engineering Cu_2_O from flat films into nanowire arrays (NWAs) to facilitate photocarrier extraction (Qu et al., 2019). By incorporating the merit of all measures, the Cu_2_O NWA-based photocathodes were manifested as the landmark featuring their excellent photoelectrochemical (PEC) efficiency and stability (Huang et al., 2013). Unfortunately, these three-dimensional architectures also drastically increased the difficulty of the fabrication processes. Given the great curvatures and existing shaded area on NWAs, only atomic-layer deposition is capable of realizing the uniform heterojunctions with intimate contact of components (Luo et al., 2016). Undoubtedly, the realization of the desired coating is the key to fully eliciting the potential of the complicated nanostructures.

Specifically, titanium oxide appears to be one of the essential components in the coating. It does not only prevent corrosions of Cu_2_O from the electrolyte solution but also rectifies the flow of photogenerated carriers (Li et al., 2015). The heterojunction between Cu_2_O and titanium oxide *via* various techniques has been demonstrated as essential for these high-performance photocathodes (Siripala et al., 2003; Lumley et al., 2019). However, as we stated, there are only extremely limited approaches that can be used for Cu_2_O NWAs (and certainly for other p-type nanostructures). Besides, titanium oxide has other uses (Azevedo et al., 2016; Yang et al., 2018; Wang et al., 2021). The most well-established yet almost the only route without post-treatments, the atomic-layer deposition technique, demands sophisticated instrumentation with expensive precursors (organometallic complexes) and experienced selection of conditions that delivers great impacts to the properties of the coating (Dai et al., 2014). On the other hand, there are incompatibility issues during the chemical depositions, whereas many of the bared p-type structures are instable. For instance, the hydrolysis of Ti alkoxide forms uniform titania coatings but requires following high-temperature annealing for the crystallization, which may be destructive for the bottom nanostructures (Paracchino et al., 2011). Therefore, a facile and mild titanium oxide deposition method is highly desired.

Specifically, there is no ready-to-use and uniform coating of semiconductive titanium oxides on Cu_2_O NWAs *via* chemical room temperature chemical route yet (Jang and Lee, 2019). The chemical limitation in aqueous electrolytes has been suggested by the Pourbaix diagram of the Cu element (Yang et al., 2017). The compatible conditions for Cu_2_O are restricted to near-neutral solutions with a narrow redox potential range. Notably, many previously reported electrodeposition of titanium oxides in harsh pH and oxidative conditions are not applicable (Eisenberg et al., 2014), for instance, the cathodic coating using the peroxide-dissolved Ti complex or anodizing Ti^3+^ in the strongly acidic solution (Kavan et al., 1993; Matsumoto et al., 2000). Nonetheless, titanium oxide layers from oxidizing Ti(III) over other nanostructures effectively formed various junctions, exhibiting outstanding photochemical or PEC properties (Toe et al., 2019). Unfortunately, none of these electrochemical processes is applicable to Cu_2_O nanostructures.

In this work, we successfully developed a new electrochemical route to realize the coating of functional titania over Cu_2_O NWAs. Owing to the mild environment and the deliberately controlled transient anodization, the coating thickness can be regulated with the well-maintained original NWA morphology. More importantly, the great portion of Ti^3+^ in the structure directly endows the coating's good charge extraction and transport ability. Specifically, the almost tripled photocurrent (from 1.17 to 3.07 mA/cm^2^, 0.55 V *vs*. RHE) and the positive shift of onset potential of the Cu_2_O–TiO_x_ NWA photocathodes clearly prove the formation of a heterojunction facilitating the directional flow of photoelectrons. Moreover, combining its functionality with the assistance of the HER catalyst further raises the activity of the photocathodes. These features of the photocathodes present a new promising route of using a rationally designed electrochemical process to fabricate uniform and functioning heterostructure under mild conditions.

## Results and Discussion

**FIGURE 1:**
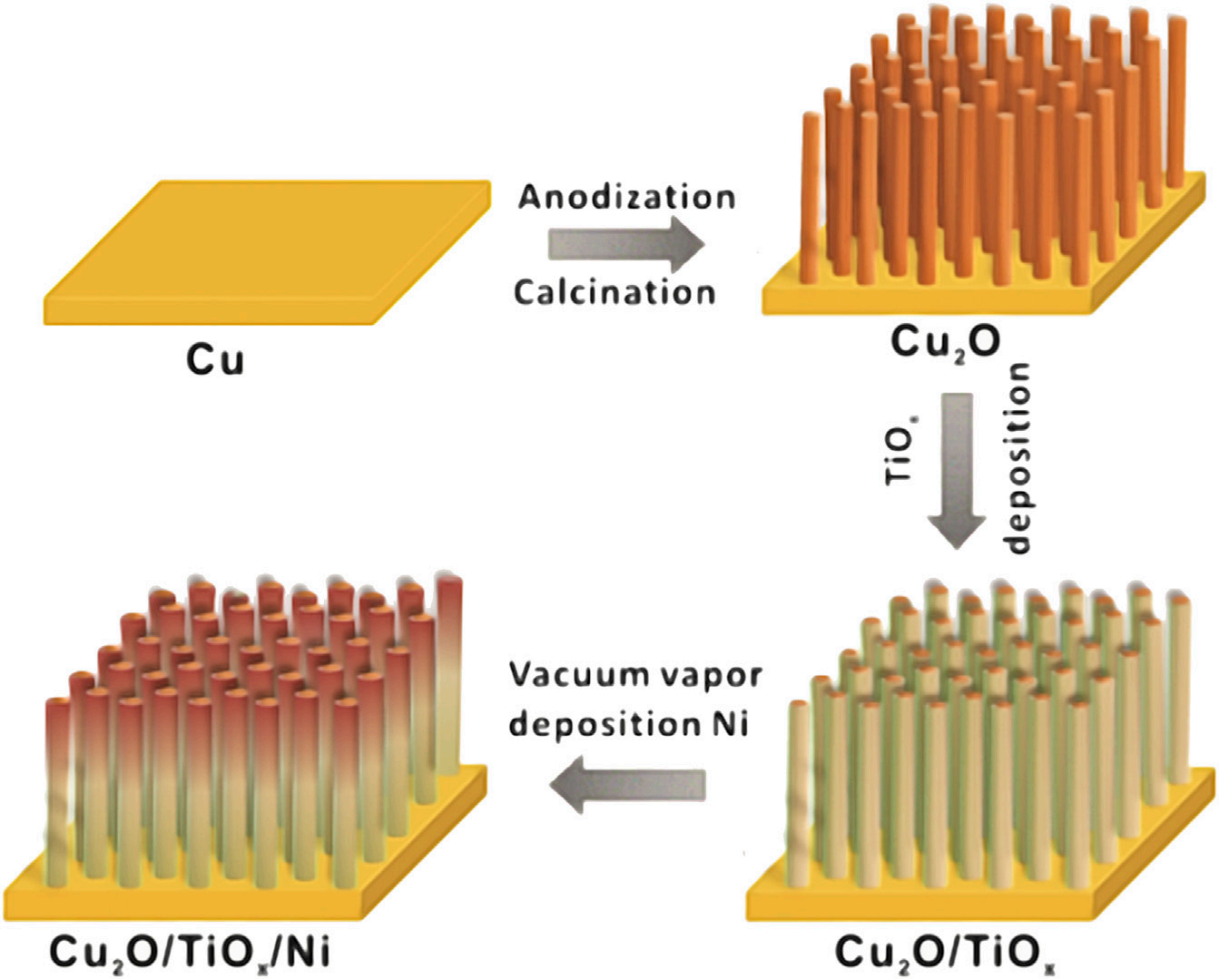
Schematic illustration of synthetic procedure of Cu_2_O/TiO_x_/Ni-NWAs heterostructure film.

**FIGURE 2:**
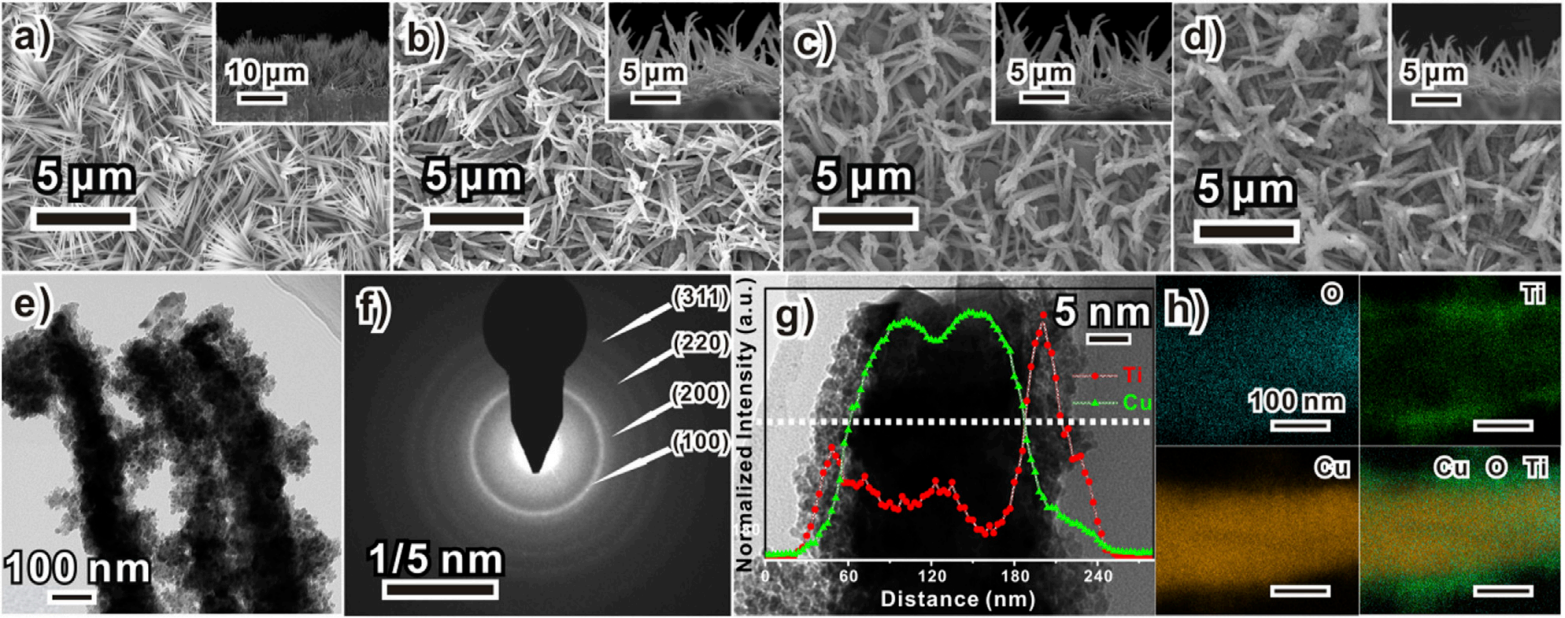
Morphology of Cu_2_O/TiO_x_/Ni composite electrode formation process of a sample **(A)** Cu(OH)_2_ NWA, **(B)** bare Cu_2_O NWA, **(C)** Cu_2_O/TiO_x_, and **(D)** Cu_2_O/TiO_x_/Ni (insets are their corresponding cross-section images) NWA; **(E)** TEM image and **(F)** selected area electron diffraction pattern of Cu_2_O/TiO_x_ NWs; **(G)** HRTEM high magnification images of Cu_2_O/TiO_x_ NW with its line-scanned element distribution profile and **(H)** elemental mapping of O, Ti, and Cu.

Figure 1 schematically illustrates the preparation process of the Cu_2_O/TiO_x_/Ni NWA photocathodes. The bottom Cu_2_O NWAs were prepared by a two-step method using our previously reported anodization process for uniformed Cu(OH)_2_ NWAs, followed by a spontaneous thermal conversion to Cu_2_O in an inert atmosphere (Cao et al., 2020). Morphologies of the photocathode at different fabrication stages were shown in scanning electron microscope (SEM) graphs (Figures 2A–D). The process was initiated by preparing uniform high-density Cu(OH)_2_ NWAs, with an average length of approximately 9 μm. After annealing in N_2_, Cu(OH)_2_ was converted into Cu_2_O NWAs. As seen from Figure 2B, the transformed arrays consisted of twisted and slightly shortened nanowires with an average length of 6.8 μm. The typical obtained Cu_2_O/TiO_x_ NWAs are shown in Figure 2C. The thin layer of amorphous TiO_x_ can barely be seen on the SEM micrograph, whereas some of the adjacent NWs were seized as bundles by the coating. The thickness of the TiO_x_ layer was estimated using transmission electron microscope (TEM) (Figure 2E) and high-angle annular dark-field scanning transmission electron microscope/energy dispersive X-ray mapping (Figure 2H) as approximately 30–50 nm. The aspect ratio slightly decreased but still could provide shortened diffusion paths for the photoelectron to the solid-electrolyte interface than the flat structures. Eventually, a thin layer (∼3 nm) of Ni was deposited onto the structure as the hydrogen evolution reaction (HER) catalyst, with no evident morphological impact (Figure 2D).

**FIGURE 3:**
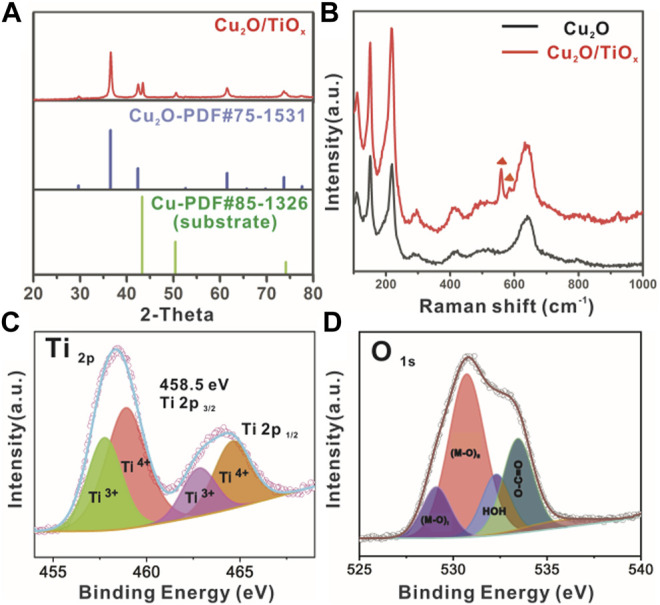
**(A)** XRD pattern of typical Cu_2_O/TiO_x_ NWA sample; **(B)** Raman spectra of Cu_2_O and Cu_2_O/TiO_x_ NWA samples; **(C,D)** X-ray photoelectron spectroscopy signal of Ti 2p and O 1s from Cu_2_O/TiO_x_, respectively.

The nature of the electrodeposited titanium oxide coating layer was revealed by TEM microscopy. Figure 2E shows the typical structure of Cu_2_O/TiO_x_ core-shell NWs in the array, clearly showing that Cu_2_O NWs were encapsulated by an amorphous layer. The titania layer was approximately 40 nm thick and assembled by primary clusters smaller than 5 nm. Micrographs showed intimate contacts of this coating layer with the inner Cu_2_O NW. A minor porosity and notable roughness can be found as well. The selected area electron diffraction pattern (Figure 2F) only exhibited rings corresponding to (111), (200), (220), and (311) d-spacing of Cu_2_O, suggesting its polycrystalline nature. No evidence of crystalized titanium oxides was observed either, which agrees with the X-ray diffraction (XRD) result (Figure 3A). High-angle annular dark-field scanning transmission electron microscope/energy dispersive X-ray mapping (Figure 2H) illustrates the elemental distribution line profile and mapping across a typical NW. The signal of oxygen sketches the overall shape of the NW, whereas Cu and Ti panels clearly confirm the spatial relationship of a core-shell structure.

The phase composition of the heterostructure was confirmed by XRD as well (Figure 3A). Despite the diffraction of 43.3 and 50.4° corresponding to (111) and (200) d-spacing of metallic Cu substrate (JCPDS 58-1326), respectively, all other peaks can be indexed to the pattern of cubic Cu_2_O (JCPDS 75-1531). No other diffraction peaks were found on the XRD pattern, suggesting the TiO_x_ layer was amorphous. Raman spectroscopy was used to further study the composition regarding the existence of amorphous oxides. Figure 3B shows the Raman spectra of Cu_2_O and Cu_2_O/TiO_x_ samples. Common features on both spectra at 109, 148, 416, 515, and 635 cm^−1^ can be assigned to the vibrational modes of Cu_2_O lattices (Maldonado-Larios et al., 2020). Different from the XRD technique, TiO_x_ (amorphous) on the Cu_2_O surface is visible to the Raman spectroscopic study. The bands at 568 and 608 cm^−1^ that emerged on the spectrum of Cu_2_O/TiO_x_ sample are associated with the deformation of out-of-plane rings (Ohsaka et al., 1978; Jumat et al., 2017). Given that no other peaks were observed, the Raman spectroscopy strongly supported the existence of titania as an amorphous form. Moreover, the successful coating was further confirmed by the ultraviolet–visible diffuse reflectance spectroscopy of Cu_2_O NWAs and Cu_2_O/TiO_x_ NWAs samples (Supplementary Figure S1). The latter had much stronger light absorption in ultraviolet, indicating the deposition of the TiO_x_ layer over the entire sample. The absorption in the visible range slightly decreased (in K-M expression), which is likely due to the increased scattering. Nonetheless, from the view of PEC efficiency, this is negligible on the scale of the reflection rates (Supplementary Figure S1).

X-ray photoelectron spectroscopy survey was carried out to collect the valence state and chemical environment information of elements in the Cu_2_O/TiOx photocathode. The full spectral range scans (Supplementary Figure S2) confirmed the elemental composition of Cu, O, and Ti. The fine scan of the Ti 2p spectrum (Figure 3C) shows the typical bands of Ti 2p_3/2_ and Ti 2p_1/2_ core levels at 458.5 and 464.9 eV, respectively. The experimental curves were well fitted with the Gaussian peaks model after Tougaard background subtraction, whereas the valence states of Ti can be identified by deconvoluting the fitting result, consisting of both Ti^3+^ and Ti^4+^. To our surprise, the percentage of Ti^3+^ is as high as 38% in the coating layer (Supplementary Table S1). The signal from O1s is slightly more complicated (Figure 3D), showing three different chemical environments: metal oxides (Ti-O at 529.1 and Cu-O 530.7 eV), hydroxyl (535.6 eV), and absorbed water (532.3 eV). Because the sample was not annealed, the carboxylic group should be the result of residue oxalate in the electrolyte. It is worthy to note that the signal from Cu in Cu_2_O is stronger and higher than the expectation of having a screening effect from the titania shell. According to TEM (Figures 2E–G), this could be from the recrystallized small Cu_2_O clusters during the deposition process. In short, the X-ray photoelectron spectroscopy result confirms the overall composition of the sample as oxides, but the coating layer cannot be simply regarded as TiO_2_, given its extremely high level of Ti^3+^. On the other hand, apparently, the coating does not contain a large number of hydroxyl groups. This can be beneficial to charge transport by reducing the chance of recombination (Carp et al., 2004).

The structural analysis discussed earlier proved the realization of our electrochemical route. Chemical compatibility is the essential challenge in this deposition process. Given the vulnerability of Cu_2_O, the electrolyte solution has to be near neutral. On the other side, Ti^3+^ is unstable as well, which hydrolyzes when pH > 4 and can be easily oxidized into hydrated TiO_2_ (Johnson et al., 2017). Hence, all reported cases were performed under very low pH with relatively stable substrates. We took several measures to stabilize Ti^3+^ and bring it to a workable condition for Cu_2_O. A water/ethylene glycol solution was used instead of a typical pure aqueous system. Hydrolysis of Ti(III) can be notably reduced with the interaction between high valent ions with the polyol environment. An optimal composition was experimentally determined as 50% (Supplementary Figure S3). Chelating/complex agents were introduced to further stabilize Ti(III) species (Lavacchi et al., 2021). The oxalic acid was selected based on both the coating coverage and photocurrent of the product.

To further achieve the uniform TiO_x_ distribution on the Cu_2_O, other important parameters were carefully evaluated as well. Surfactants have been proved beneficial in many electrodeposition systems. For this system, we found that Triton X100 could moderately improve the quality of the product (Supplementary Figure S4), which likely takes effect by facilitating the wetting and diffusion of electrolytes in the NWAs (Liu et al., 2020). Again, the deposition temperature gave almost no impact on TiO_x_ (Supplementary Figure S5), indicating that the rate-determining step in the deposition is dominated by temperature-insensitive factors.

**FIGURE 4:**
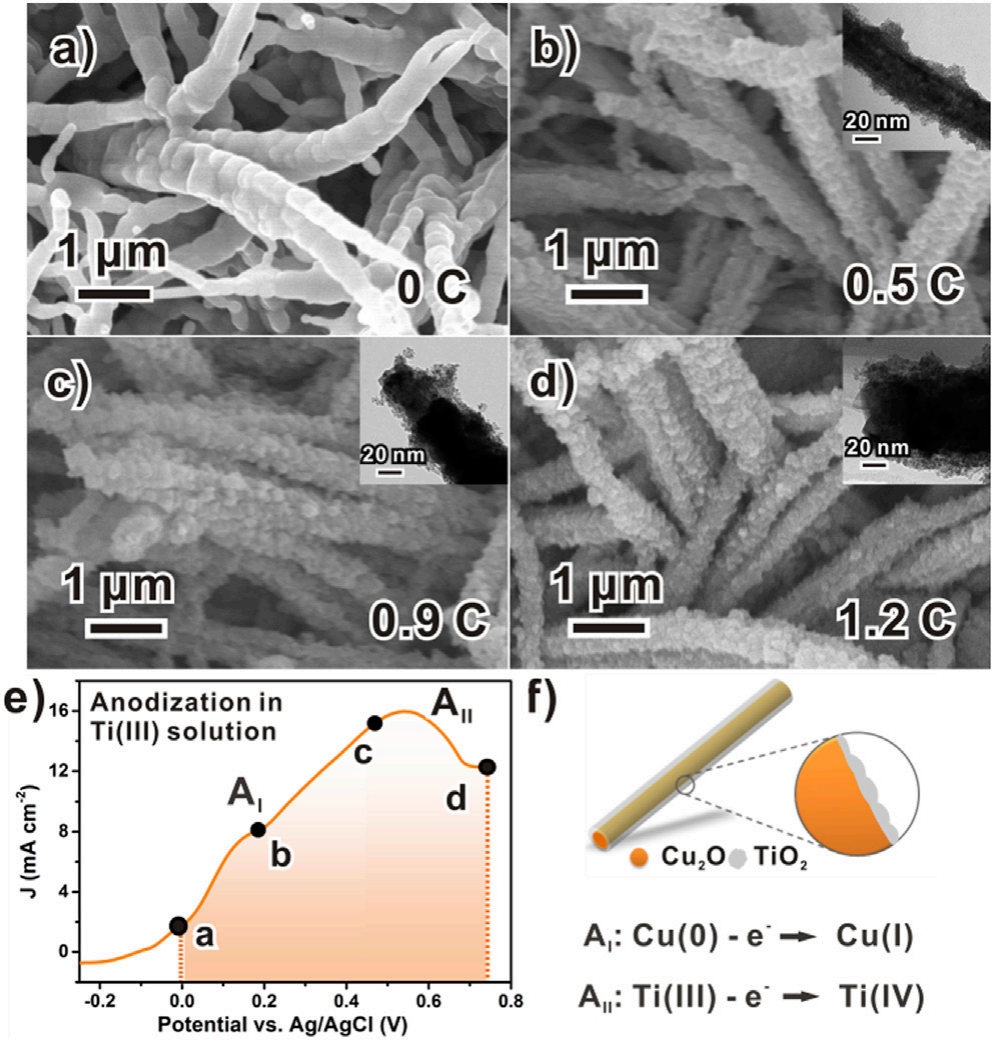
Chronology of TiO_x_ formation in potentiodynamic anodization process: **(A)** Scanning electron microscope images of bare Cu_2_O NWAs; **(B–D)** Corresponding scanning electron microscope images showing morphological evolution of amorphous TiO_x_ along with increasing anodization charge. **(E)** Typical LSV curve of anodizing a piece of Cu_2_O foil with rate of 5 mV/s, data points labeled with charges specifying samples examined in parts b–d and **(F)** schematic model to formation of Cu_2_O/TiO_x_ NWAs.

Furthermore, the typical linear sweep voltammetry (LSV) curve (Figure 4E) of this anodic reaction demonstrates more details of the process. Despite a minor side reaction from the substrate (A_I_ peak), anodic signals form one broad peak. Such a feature suggests the chemical inhomogeneity of Ti(III) species, as they should be a series of hydroxyl oligomers (Qin et al., 2020). Indeed, a minor Tindall effect was found with the deposition electrolyte “solution” (or should be called “sol”), although it is stable for several hours. The stability of the electrolyte is sufficient for us to prepare approximately six parallel samples at one time, so it has high reproducibility. The distance between the quartz electrolytic cell (10 × 10 cm^2^) and the light source is 5 cm, which needs to meet the luminous power of up to 100 mW. The working area of the electrode is 1 × 1 cm^2^.

Different electrochemical techniques were considered as well. Ideally, the electrodeposition can be performed using various techniques, such as the galvanostatic or potentiostatic process, or transient methods such as linear potential scans or AC controls. However, as we tested, none of the galvanostatic or potentiostatic processes produced reliable and reproducible results. This could be due to the large surface conductivity change during the deposition. On the contrary, directly using the LSV scan worked quite well for the deposition. In addition, the scan rate can be used as a handy tool to kinetically discriminate reactions, which reduces the redox corrosion to Cu_2_O. In our case, severe destruction to the NWA structure or insufficient deposition was observed with inappropriate scan rates (Supplementary Figure S6).

Moreover, fine control of the thickness of TiO_x_ can be achieved by regulating the anodization charge during the deposition process (Figure 4). Figure 4A shows the typical relatively smooth surface of the pristine Cu_2_O structures before the deposition of TiO_x_. After that, as the anodization progressed, amorphous TiO_x_ was gradually loaded onto the Cu_2_O surface (Figures 4B–D) with increasing amounts. At the anodic charge of 0.5 C cm^−1^ (Figure 4B), the Cu_2_O surface was roughened with the discrete coating of approximately 10 nm, which is not sufficient to completely hinder the photo-corrosion of Cu_2_O yet. Meanwhile, a minor improvement to the photocurrent was observed (Supplementary Figure S7). As the charge increased to 0.9 C cm^−1^, more amorphous TiO_x_ was loaded onto Cu_2_O, whereas the thickness was measured as approximately 20 nm (Figure 4C). In the finishing stage, the Cu_2_O NWs were fully covered by the deposited TiO_x_ layer, with the coating thickness eventually reaching 40 nm (Figure 4D). The complete deposition notably improved water reduction photocurrents of the NWA photocathode from 1.35 to 4.15 mA/cm^2^ at 0.55 V *vs*. RHE (Supplementary Figure S7). In principle, further thicker coating might be possible by optimizing conditions. However, for our current purpose in the PEC application, coating less than 50 nm is reasonable and similar to the typical thickness of reported titanium oxide layers (Musselman et al., 2010).

**FIGURE 5:**
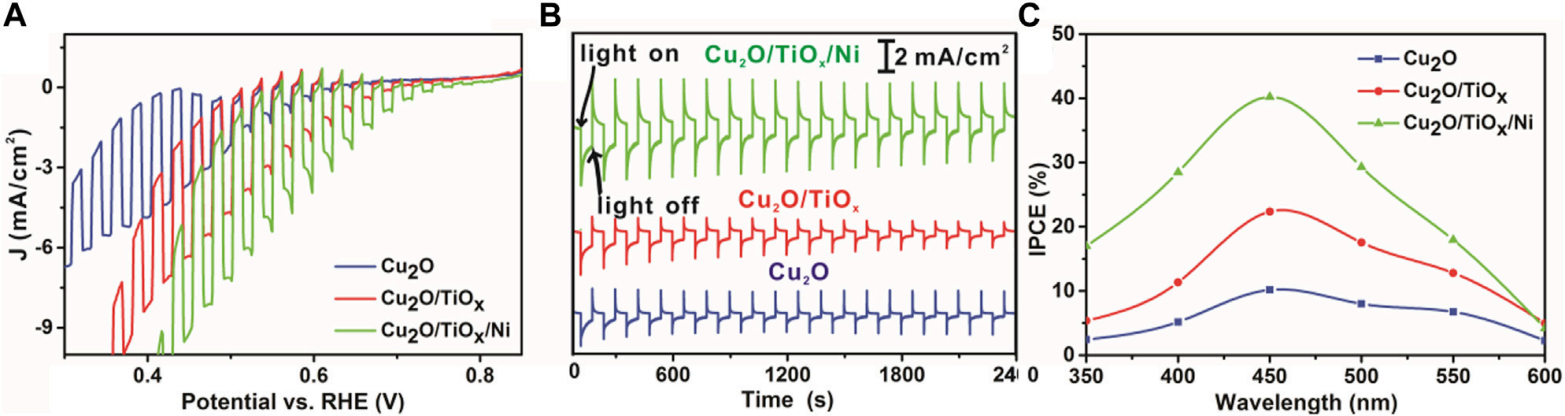
Current-potential (I–V) characteristics curve, stability, and IPCE test **(A)** I–V curves under chopping simulated AM 1.5G illumination **(B)** stability measurements with constant bias at 0.6 V *vs*. RHE and under simulated AM 1.5G illumination **(C)** IPCE of pristine Cu_2_O NWAs, Cu_2_O/TiO_x_ NWA, and Cu_2_O/TiO_x_/Ni NWA samples (all tests performed in pH = 6 electrolytes).

The PEC performance confirmed the functionality of the coating layer, as shown in Figure 5. Specifically, LSV scans of pristine-Cu_2_O, Cu_2_O/TiO_x_, and Cu_2_O/TiO_x_/Ni photocathodes were collected in 0.5-M sodium phosphate buffer under chopping AM1.5G illumination. The current density–potential curves clearly show that Cu_2_O/TiO_x_ photocathode had a significantly enhanced photoactivity, in which photocurrent density reached −3.0 mA/cm^2^ at 0.55 V *vs*. RHE, whereas the pristine-Cu_2_O only had -1.1 mA/cm^2^ parallelly. Moreover, with Ni cocatalyst loaded onto Cu_2_O/TiO_x_ photocathodes, the photocurrent density could be further enhanced to −4.7 mA/cm^2^ at 0.55 V *vs*. RHE, which is over four times higher than that of pristine Cu_2_O photocathode, showing a significant enhancement in photo-response that resulted from the effective heterojunction formed inside of Cu_2_O/TiO_x_. Furthermore, Cu_2_O/TiO_x_/Ni photocathode had a more positive onset potential of 0.82 V *vs*. RHE, 0.18 V positive to the Cu_2_O photocathode, as shown in Figure 5A. Both of these phenomena indicate that the Cu_2_O/TiO_x_/Ni interface could significantly improve the utilization of photocarriers with the smaller hindrance and the lower activation energy barrier in comparison with the pristine Cu_2_O surface.

As is shown in Figure 5B, the TiO_x_ coating and Ni cocatalyst notably benefited the stability of the photocathodes. The photocurrent of pristine-Cu_2_O photocathode decreased by approximately 60% in 40 min. SEM (Supplementary Figure S8) image as pieces of evidence that the NWs were seriously damaged and deformed. Energy dispersive X-ray (EDX) analysis confirms the decrease of oxygen content, indicating that the Cu_2_O was considerably reduced. This agrees with the typical photo-corrosion mechanism of Cu_2_O (Toe et al., 2019). With the coating of the TiO_x_ layer, the stability of the photocathode was notably improved. The photocurrent density decreased by approximately 40%. It is reasonable to speculate that it was still due to the self-reduction reaction, according to the EDX result. Metallic Cu nanoparticles can be distinctly discriminated after the test (Supplementary Figure S8D), implying the leakage of Cu to the electrolyte. Furthermore, the location of the speciated Cu islands indicates that the photoelectrons have been extracted to the TiO_x_ part. Otherwise, the reduction of Cu_2_O should not produce Cu crystallites outside the coating. Nonetheless, HER was not the favored reaction over the surface due to lacking HER sites. Once the necessary catalyst (Ni) was integrated, the photocathodes became much more stable with better PEC efficiency. SEM and EDX (Supplementary Table S2) confirmed that the morphology and composition of Cu_2_O/TiO_x_/Ni photocathode were well maintained after the stability test. Only very few bright metallic Cu islands can be found on the surface of the photocathode, which validated the functionality of the cocatalyst. It is worthy to emphasize that the coating of this single TiO_x_ layer is helpful for the photoelectron collection from Cu_2_O but still not the ultimate solution to the system. Clearly, an additional measure is necessary to fill out the pores, and the efficiency of the photocathodes can be further improved. Nonetheless, the deposition of TiO_x_ solved one of the most important yet challenging steps for the functionalization of Cu_2_O.

The key advantage of the nanostructure approach is the ability to improve the charge transport and minimize the recombination by constructing heterojunction and providing a stable support structure for the cocatalyst. The incident photon-to-current efficiency (IPCE) obtained from the LSV measurement was greatly improved. As shown in Figure 5C, the IPCE for the Cu_2_O/TiO_x_/Ni photocathode is reasonably higher than that of the pristine-Cu_2_O across the whole wavelengths, especially in the visible region between 420 and 470 nm.

**FIGURE 6:**
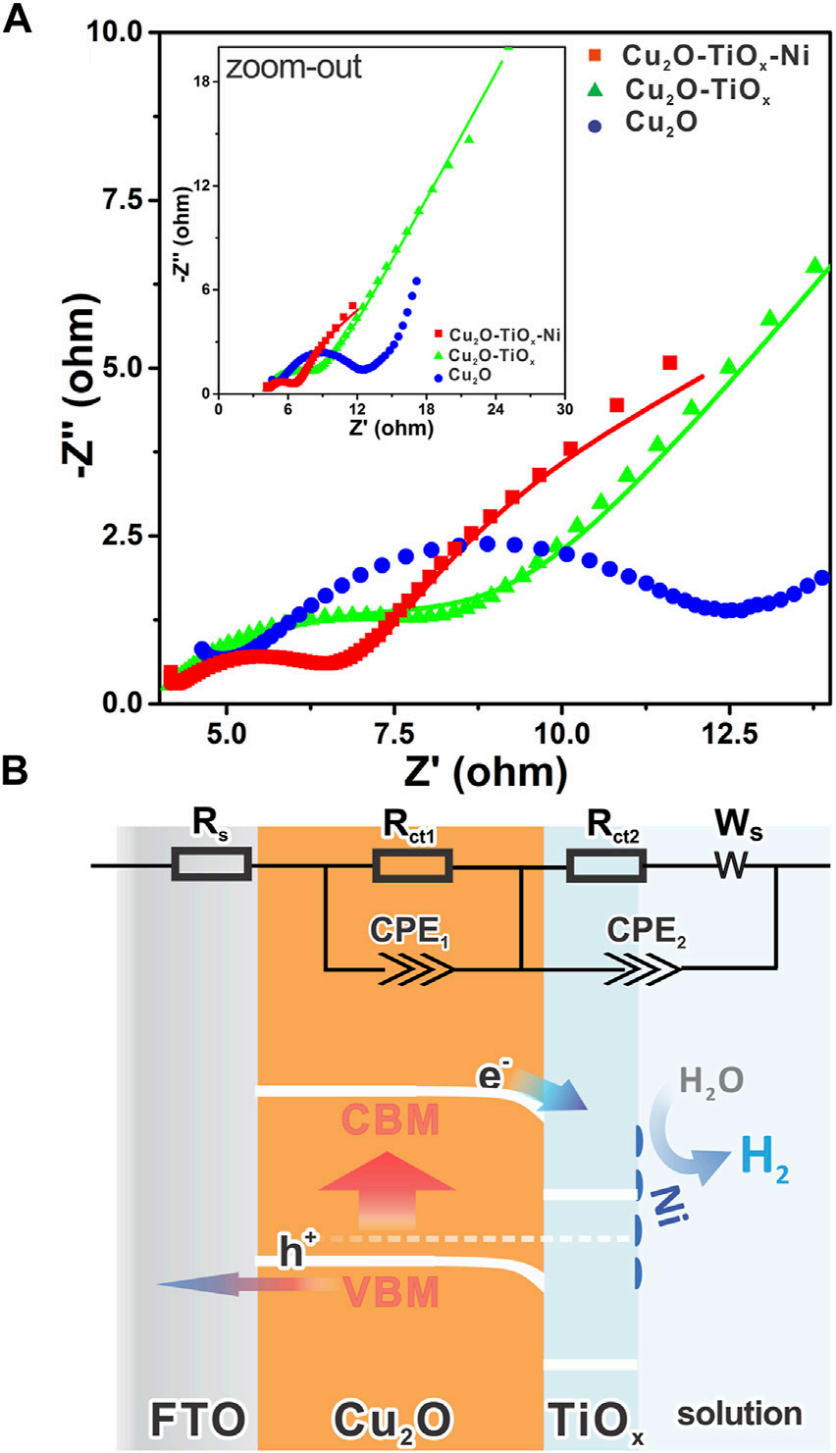
**(A)** Nyquist plots of different electrodes in 0.5 M phosphate buffer under light irradiation (λ = 455 nm) with electrochemical impedance spectroscopy at a DC potential of 0.5 V *vs*. Ag/AgCl in AC potential frequency range of 10 to 1 Hz, inset shows zoomed-out view with fewer details; **(B)** schematic band diagram of Cu_2_O/TiO_x_/Ni photocathode representing internal charge transfer direction, with inset showing equivalent circuit for electrochemical impedance spectroscopy analysis.

To understand the details of the charge transfer in the photocathodes, we performed an electrochemical impedance spectroscopy (EIS) study, which is a powerful frequency domain analysis technique for investigating electrical characters of interfaces (Chen et al., 2017). Figure 6A shows the impedance data of parallelly tested samples with complex coordinates as Nyquist plots. The very high-frequency region on the lower left corresponds to the series resistance and response from the counter electrode, which is approximately 5 Ω for all samples. Semicircles on the plots represent the impedance of interfaces or electrochemical processes, whereas the diameters of semicircles are equal to the corresponding resistances. Straightforwardly, a larger semicircle correlates to worse charge transportation through that interface. The linear portion of the lower frequency, so-called a Warburg element, represents the diffusion-controlled impedance (Kecsenovity et al., 2017). Visually, the charge transfer difference of Cu_2_O, Cu_2_O/TiO_x_, and Cu_2_O/TiO_x_/Ni photocathodes can be clearly addressed in the plots (Figure 6A). The step-by-step architecting by the TiO_x_ coating and cocatalyst loading gradually lowered the overall charge transport impedance. The fitting result (Supplementary Table S3) indicates that the Cu_2_O/TiO_x_ samples have a much smaller *R*_*ct2*_ in comparison with pristine-Cu_2_O, which benefits from the promoted charge separation and transport under the assistance of the Cu_2_O–TiO_x_ heterojunction. Meanwhile, *CPE1* has increased several times, implying a capacitive feature on the Cu_2_O–TiO_x_ interface. All interfacial resistances were further reduced by Ni cocatalyst loading. Particularly, *CPE2* is notably smaller, suggesting less charge accumulation on the photocathodes because of the expedited HER pathway.

**FIGURE 7:**
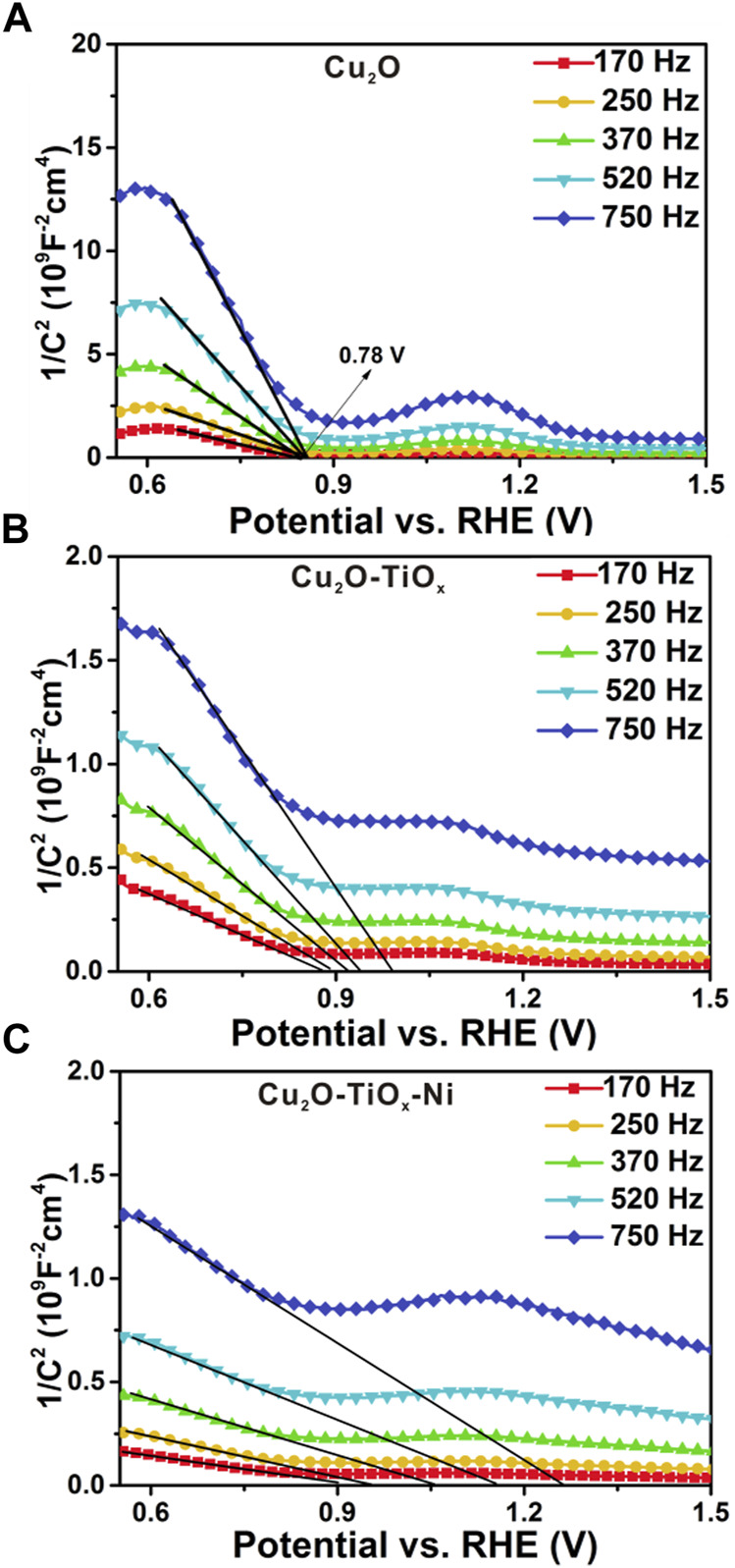
Mott–Schottky plots **(A–C)** for bared Cu_2_O, Cu_2_O/TiO_x_, and Cu_2_O/TiO_x_/Ni photocathodes, respectively. Measurements performed from 0.0 to 1.0 V *vs.* Ag/AgCl in pH = 6.0 0.5 M potassium phosphate buffer using multi-sine staircase potential impedance technique.

Figures 7A–C show the Mott–Schottky (M-S) measurement results of the photocathodes. The measurement can be used to determine the type of semiconductors, flat band potentials, and carrier densities (Patel et al., 2017; Sivula, 2021). Using the following equation, important semiconductor parameters were linked together:

$$\frac{1}{C_{SC}^{2}}=\frac{2}{eN\varepsilon \varepsilon_{o}A^{2}}\left[\left(V-V_{fb}\right)-\frac{Tk_{B}}{e}\right]$$

where *C*_*SC*_ is the space-charge capacitance, *V* is the applied potential, *V*_*fb*_ is the flat band potential, *ε* is the dielectric constant of Cu_2_O, *ε*_*0*_ is the permittivity of vacuum, *A* is the area, *N* is the carrier density, *e* is the electron charge, *k*_*B*_ is the Boltzmann’s constant, and *T* is the absolute temperature.

According to the equation discussed earlier, intercepts of the curve in M-S plots were used to determine *V*_*fb*_. For the TiO_x_ (Supplementary Figure S9) on metal and the bared Cu_2_O NWA (Figure 7A, a relatively straightforward result can be obtained indicating their n-type and p-type semiconductive nature, respectively. However, in the case of the possible frequency dispersion caused by nonideal behaviors of electrode capacitances, their M-S correlations were plotted using a series of frequencies. The dataset suggests the *V*_*fb*_ of the Cu_2_O photocathode as 0.78 V *vs*. RHE. For Cu_2_O/TiO_x_ and Cu_2_O/TiO_x_/Ni photocathodes, large frequency dispersion was observed. A precise determination of their flat band potentials is unrealistic, although their *V*_*fb*_ can be speculated to be similar to pristine-Cu_2_O or with minor positive shifts. On the other hand, the measurements manifest the carrier density difference between the three electrodes. Specifically, the reciprocal of slopes on the tangents are proportional to *N*, the carrier densities in the space charge region. Despite the frequency dispersion, the slopes of Cu_2_O/TiO_x_ are an order smaller than pristine-Cu_2_O, suggesting more responding charge carriers in the space charge region. This increment hints at a more efficient extraction of photoelectrons and less recombination in the bulk. According to the structure of the photocathodes, this beneficial effect shall be attributed to the electric field formed on the heterojunction interface, which alters the carrier distribution in Cu_2_O and subsequently notably facilitates the carrier extraction.

## Conclusion

In short, we successfully achieved the aimed titania electrodeposition in near-neutral conditions for preparing uniform heterojunction over a vulnerable p-type semiconductor nanostructure. Due to the existence of a large portion of Ti^3+^, this electrodeposited layer directly exhibited a charge transport ability without any post-treatment. Both the onset potential and PEC efficiency of the NWA photocathodes were greatly improved. By further incorporating with Ni-based HER cocatalyst, the accomplished photocathodes exhibited a photocurrent approximately four times of the pristine-Cu_2_O NWAs, with a much longer lifetime. Electrochemical impedance spectroscopy and M-S measurements revealed that the TiO_x_ layer facilitated the charge transport inside the space charge region. This protocol is reliable and effective but requires no sophisticated instrumentation. These merits make it easily applicable to other unstable nanostructures. Moreover, these results demonstrated the possibility of electrochemically fabricating cost-effective photoelectrodes with both earth-abundant materials and affordable preparation expenditures.

## Data Availability

The original contributions presented in the study are included in the article/Supplementary Material; further inquiries can be directed to the corresponding authors.
