# Supplementary material for: Room Temperature Electrodeposition of Ready-to-Use TiOx for Uniform p-n Heterojunction Over Nanoarchitecture
Source: Front Chem. 2022 Feb 22;10:832342. doi: 10.3389/fchem.2022.832342 (PMC8902498; doi:10.3389/fchem.2022.832342)
Supplement: Supplementary file 1 [file DataSheet1.PDF]

Supplementary Material

Experimental

All chemicals used in this study were analytical grades and used without further purification. The copper foil (99.9%, 1.0 mm thick, 1×1 cm^2) was cleaned with acetone, ethanol and deionized water in turn.

(1) Preparation of Cu_2O NWA Photocathodes

The copper foil was anodized in an electrochemical cell with a typical standard three-electrode setup, using an alkali solution (3 M KOH and 0.07 M cetyltrimethylammonium bromide (CTAB)) to form $Cu(OH)_2$/Cu NWAs foil. The Cu_2O/Cu foil formed by a thermal treatment which was carried out with a heating ramp of 5 °C min^{-1} and maintained at 350 °C for 1 h in N_2 atmosphere. Afterward, the samples were left to cool naturally to room temperature and then collected for further treatment.

(2) Preparation of Cu_2O/TiO_x NWA Photocathodes

The Cu_2O/TiO_x NWA Photocathodes were prepared by potentiodynamic anodization with the three-electrode configuration (a Pt wire as the counter electrode and Ag/AgCl as the reference electrode). The electrolyte solution was made of 1.26 g oxalic acid, 2.5 mL $TiCl_3$ (18%), 50 mL ethylene glycol and 50 mL of deionized water, with pH adjusted to 6 by 1 M $NaHCO_3$ solution. The potential of the work electrodes (Cu_2O NWA) was anodically scanned from -0.25 to 0.75 V (vs Ag/AgCl) at 5 mV/s. Afterwards, the Cu_2O/TiO_x photocathodes were rinsed with DI water and dried in the desiccator at room temperature.

(3) Preparation of Cu_2O/TiO_x/Ni Composite Photocathode

A layer of Ni with a thickness of 3 nm was deposited on the Cu_2O/TiO_x NWA samples by thermal evaporation to act as a co-catalyst for hydrogenation.

Photoelectrochemical and Electrochemical Impedance Measurements

Photoelectrochemical measurements were carried out on CHI-600D in a three-electrode configuration, with samples as the working electrode, a Pt wire as CE and Ag/AgCl/saturated-KCl as RE. 0.5 M aqueous phosphate solution (pH 6.0) was used as the electrolyte. In typical photocurrent tests with the Linear Sweep Voltammetry (LSV), a cathodic 5 mV/s scan was used with AM1.5G illumination (100 mW/cm^2) chopped every 5 s. The electrochemical impedance spectroscopy (EIS) measurement was carried out using the same apparatus and recorded by a Modulab-XM Solartron potentiostat using multi-sine mode and 455 nm LED (39 mW/cm^2). The frequency ranges were measured from 1 MHz to 1 Hz with a sinusoidal potential perturbation of 10 mV with zero bias to the open circuit potentials of the samples. The Mott−Schottky plots were extracted from the datasets of staircase-potential EIS measurements covered the varied potential and frequencies.

Instrumentation

The morphologies of samples were recorded by the Scanning Electron Microscope (FEI Quanta FEG 250 and Hitachi S-4800 equipped with TLD) and TEM (Tecnai F20). The chemical composition was determined by the SEM integrated Energy Dispersive Spectrometer (EDS). XRD patterns were acquired with a Bruker D8 ADVANCE DAVINCI X-ray diffractometer (Cu Kα1, 40 kV / 200 mA). The X-ray photoelectron spectroscopy (XPS) data was collected using a PHI Quantum 2000 XPS system (Al Kα source) and calibrated with adventitious C1s signal. The UV−vis Diffuse Reflectance Spectra (UV−vis DRS) were recorded with PerkinElmer Lambda 950 spectrophotometer using a Teflon microbeads reference. Raman spectra were collected using a Renishaw Raman system with Ar (532.8 nm) laser excitation.

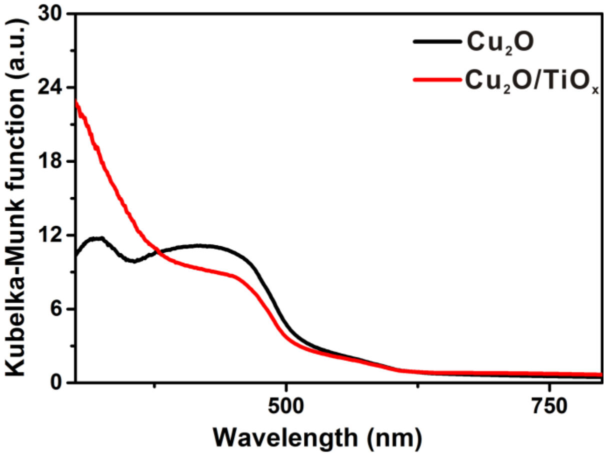

Fig. S1. Reflection spectra derived from diffuse reflectance via Kubelka-Munk theory.

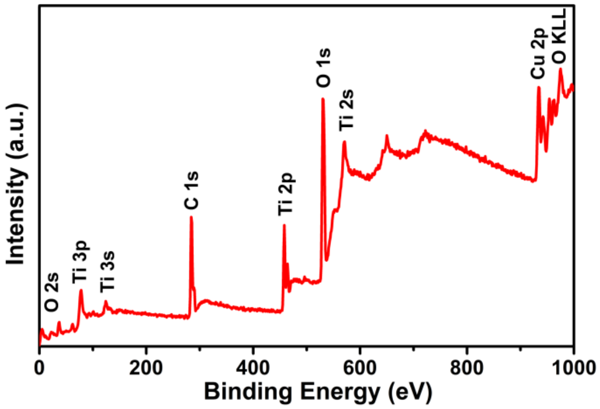

Fig. S2. XPS broad scan spectra of the typical Cu_2O/TiO_x NWA sample.

Table S1. XPS data fitting result details of Ti2p and O1s core levels.

	Area(%)	Fwhm	Position
	57698.3 (61.38)	2.58	458.9
		2.37	464.6
	36305.2 (38.62)	2.17	457.8
		2.13	462.8
	**Area**	**Fwhm**	**Position**
	99868.2	2.30	530.7
	2946.0	1.81	532.3
	48626.8	1.93	533.4
	24674.9	1.80	529.1
	9070.8	4.40	535.6

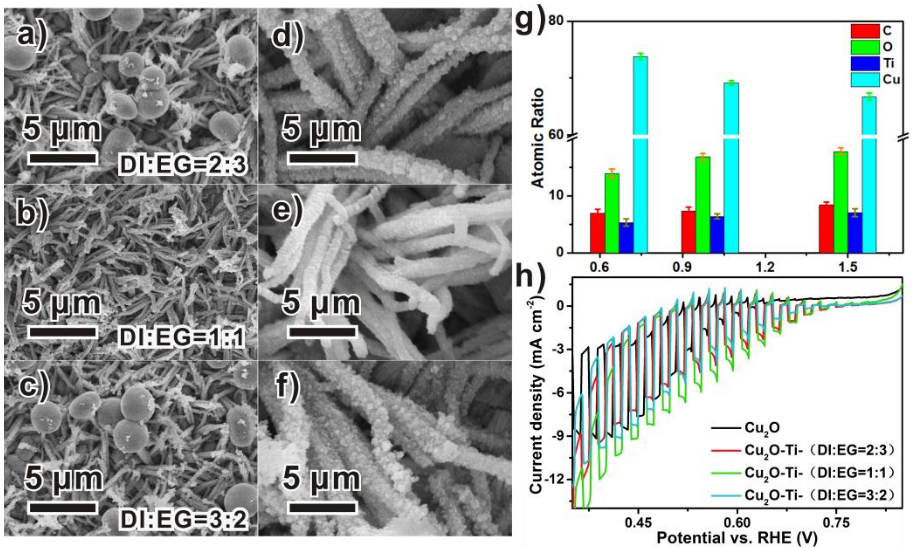

Fig. S3. Impacts of the ratio of deionized water (DI) to glycol (GR) in the anodization electrolyte solution: SEM images of Cu_2O/TiO_x NWAs prepared in (a) DI:GR=2:3, (b) DI:GR=1:1, (c) DI:GR=3:2 (d-f) enlarged images of their left images, respectively, (g) apparent surface oxygen, copper, and titanium to carbon atom ratios of anodized samples (calculated from SEM-EDS) and (h) the PEC-LSV scan (5 mV s^{-1}) curves performed in a 0.5 M potassium phosphate buffer at pH=6.0, under 36 mW cm^{-2} 455 nm LED illumination chopped every 5 s.

All of the following photocurrent and voltage (PEC-LSV) test conditions are identical and unless further specified.

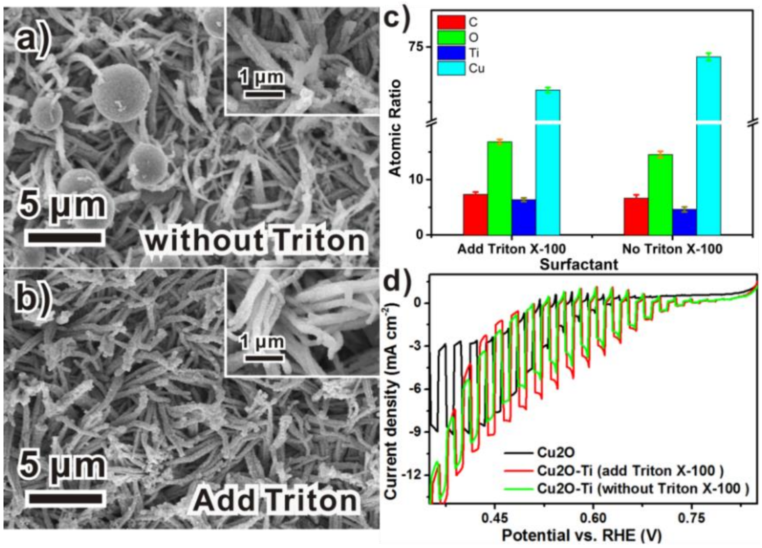

Fig. S4. Impacts of the presence or absence of surfactants (Triton X-100) in the anodization electrolyte solution: SEM images of Cu_2O/TiO_x (a) presence and (b) absence of 1 mM surfactants during the anodized deposition of TiO_x system respectively; (c)Apparent surface oxygen, copper, and titanium to carbon atom ratios of anodized samples (calculated from SEM-EDS) and (h) the PEC test of a group samples under the corresponding conditions, using default procedures as above with 455 nm blue LED.

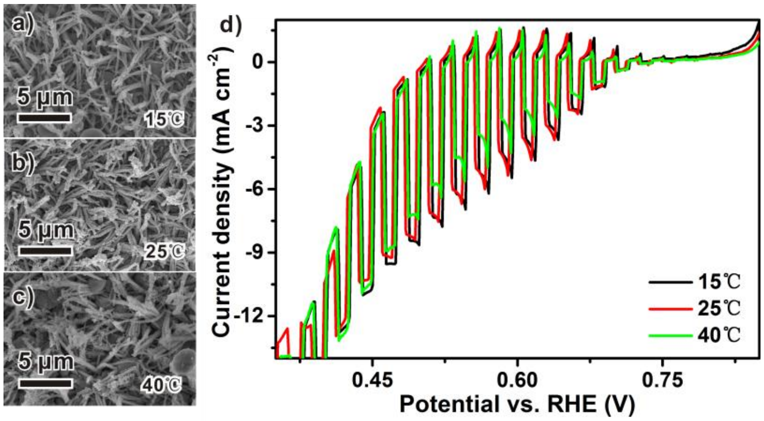

Fig. S5. Impacts of temperature of TiO_x deposition in the anodization electrolyte solution: SEM images of Cu_2O/TiO_x NWAs prepared in (a) 15°C, (b) 25°C, (c) 35°C and (d) the PEC test of the samples under the corresponding conditions, using default procedures as above with 455 nm blue LED.

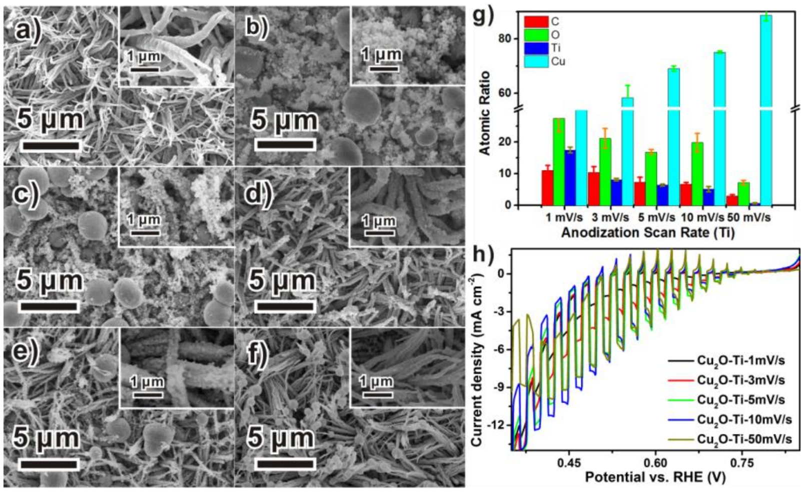

Fig. S6. Scan rate dependence of TiO_2 formation in the potentiodynamic anodization process: (a) SEM images of bare Cu_2O NWAs; Corresponding SEM images showing the morphological evolution of amorphous TiO_x along the increasing anodization scan rate (b) 1 mV/s, (c) 3 mV/s, (d) 5 mV/s, (e) 10 mV/s and (f) 50mV/s. (g) Apparent surface oxygen, copper, and titanium to carbon atom ratios of anodized samples (calculated from SEM-EDS) and (h) the PEC test of a group samples.

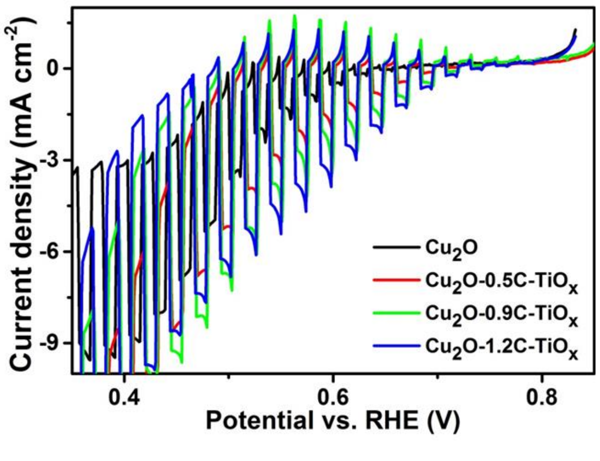

Fig. S7. Effect of the anodization charge to the TiO_x coating as controlled by stopping the anodization deposition under the default anodization rate (5mV/s).

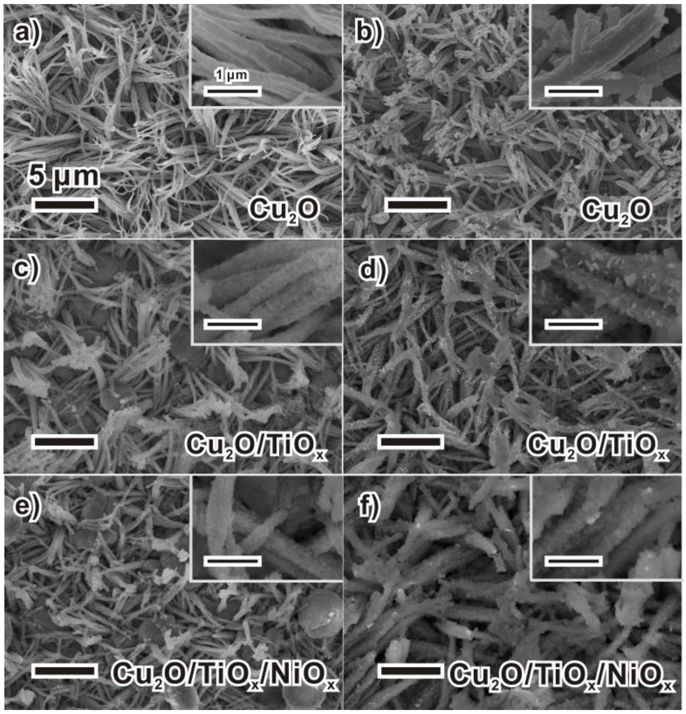

Fig. S8. SEM images of samples before and after photoelectrochemical stability testing Cu_2O-based with different photocathodes (a-c) Cu_2O, Cu_2O/TiO_x and $Cu_2O/TiO_x/Ni$ before testing; (d-f) Cu_2O, Cu_2O/TiO_x and $Cu_2O/TiO_x/Ni$ after testing.

Table S2. Apparent surface oxygen, copper, and titanium to carbon atom ratios of samples before and after photoelectrochemical stability testing with the different Cu_kO-based photocathodes (calculated from SEM-EDS).

	**Cu_2O**		**Cu_2O/TiO_x**		**$Cu_2O/TiO_x/Ni$**	
**Atomic %**	before	after	before	after	before	after
**Cu**	71.9	76.6	59.81	61.08	58.50	59.45
**O**	28.1	23.4	37.85	36.73	38.01	37.63
**Ti**	-	-	2.34	2.19	2.65	2.21
**Ni**	-	-	-	-	0.84	0.71

Table S3. Model Parameters of the EIS Results (Figure 6a).

**parameter**	**Cu_2O**	**Cu_2O/TiO_x**	**$Cu_2O/TiO_x/NiO_x$**
**R_{ss} (Ω)**	5.708	4.884	3.202
**R_{ct1} (Ω)**	6.583	5.015	4.069
**W_s-R (Ω)**	3.231	3.621	4.250
**W_s-T (x10^{-3} F cm^{-2})**	1.010	3.501	10.10
**CPE1-T (x10^{-3} F cm^{-2})**	1.454	5.261	2.332
**CPE1-P**	0.61	0.49	0.50
**R_{ct2} (Ω)**	46.52	11.07	8.501
**CPE2-T (x10^{-3} F cm^{-2})**	132.50	110.11	70.53
**CPE2-P**	0.78	0.81	0.97

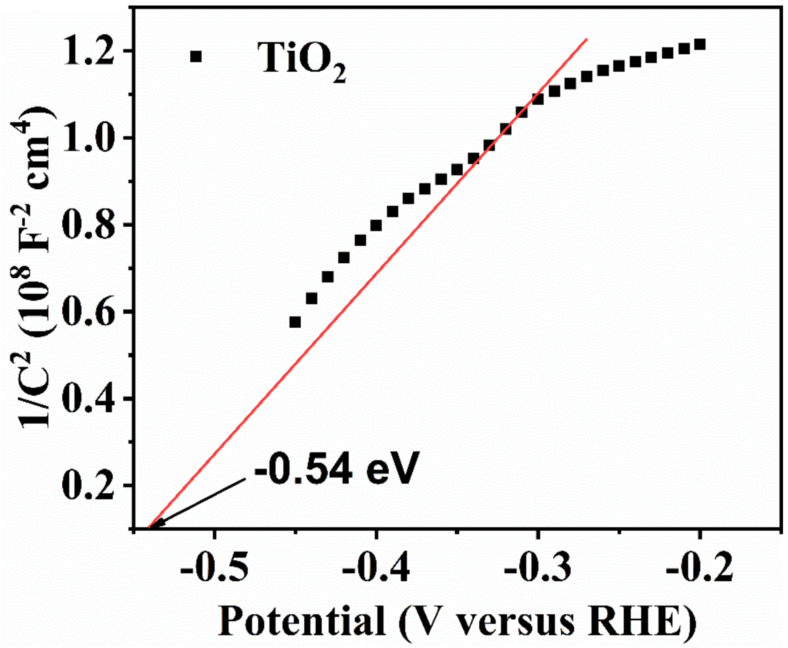

Fig. S9. M-S measurement of pure TiO_x layer on a nickel foam showing its n-type semiconductive feature (performed in a 0.5 M sodium sulfate solution, using the typical 3-electrodes configuration).
